# Impact of Reactive Temperament on Bull Fertility in Natural Service

**DOI:** 10.1111/rda.70324

**Published:** 2026-10-27

**Authors:** Larissa de Almeida Martin, Mariana Karla Francolino da Silva, Marcelo Sant’Ana Borges, Luana Gomes Fernandes, Richardt Gama Landgraf, Fábio Morato Monteiro, André Maciel Crespilho

**Affiliations:** ^1^ Santo Amaro University, UNISA São Paulo Brazil; ^2^ Department of Animal Reproduction University of São Paulo (USP) São Paulo Brazil; ^3^ Institute of Animal Science Sertãozinho Brazil; ^4^ São Paulo State University Jaboticabal Brazil; ^5^ Department of Pharmaceutical Sciences Federal University of São Paulo, UNIFESP Diadema Brazil; ^6^ Central Bela Vista Botucatu São Paulo Brazil

**Keywords:** bull, fertility, natural service, reactiveness, semen quality

## Abstract

The study aimed to evaluate the influence of temperament on semen quality and fertility in Nelore bulls. Two groups were selected based on ethological analysis, consisting of animals with either calm temperament (CA, *n* = 8) or reactive temperament (RE, *n* = 8). All bulls underwent two semen collections for sperm evaluation (pre‐ and post‐thawing), as well as serial blood collections for testosterone and cortisol measurements. Ejaculates recovered from the three most reactive and the three least reactive bulls were pooled to form two semen batches, which were subsequently cryopreserved and used for fixed‐time artificial insemination (FTAI) to evaluate the fertility of semen from bulls with the two different temperament profiles. Additionally, each bull was housed in individual paddocks with cyclic cows (average of 20 cows per bull) for 90 days for a natural service capacity test. No differences were observed in sperm kinetic parameters, morphology and plasma membrane integrity for fresh or post‐thaw semen when comparing the two experimental groups. Likewise, no effect of reactivity was observed on the fertility of calm and reactive bull semen in FTAI (*p* > 0.05). CA bulls showed lower average cortisol concentrations compared to the RE group during the second experimental routine, but there were no differences in testosterone values. CA bulls achieved higher conception rates in natural service (84.56%) compared to reactive animals (76.41%; *p* < 0.0001). In conclusion, no significant effects of temperament on fresh or post‐thaw semen quality parameters were detected in Nelore bulls under the conditions of this study. However, bulls with a calmer temperament exhibited higher reproductive performance in natural service programs, supporting the hypothesis that behavioural mating efficiency rather than intrinsic gametic failure influences pasture breeding success.

## 1 Introduction

Stress can be defined as a reflex reaction revealed by an animal's inability to cope with its environment. Some undesirable stimuli do not necessarily cause pain; however, they increase the feeling of fear, impacting stress levels and animal behaviour (Grandin 1997).

Situations that expose animals to well‐being alterations, such as stressful stimuli, lead to the release of corticotropin‐releasing hormone (CRH), which, in turn, stimulates the release of pituitary adrenocorticotropic hormone (ACTH) (Kumar et al. 2012). Acting systemically, ACTH stimulates the adrenal gland to release cortisol and catecholamines, generating rapid alert, fight or flight responses in the animal and disrupting homeostasis (Cooke et al. 2019; Friedrich et al. 2015). Additionally, increases in cortisol levels affect hypothalamic release of GnRH, pituitary release of LH and FSH, directly influencing gonadal function due to the lack of gonadotrophic stimulation for steroid hormone production (Breen and Karsch 2004; Kumar et al. 2012).

Several studies involving the human species have demonstrated that neuroendocrine changes related to stress can exert influence on testosterone production by Leydig cells, negatively affecting spermatogenesis, sperm quality and the pregnancy rates (Llacqua et al. 2018; Janevic et al. 2014).

In this context, studies involving livestock animals have also demonstrated the influence of temperament and stress on body development (Vedovatto et al. 2021), age at puberty (Cooke et al. 2019), follicular development (Guerson et al. 2021) and the fertility of female cattle (Rueda et al. 2015). Reactive male cattle also show lower weight gain rates, higher serum cortisol levels and reduced testicular thermoregulatory capacity (Braz et al. 2020), factors that can negatively impact reproductive performance. The aim of this study is to assess the effect of temperament on the quality and fertility of *Nelore* bull semen, testing the hypothesis that animals with calmer behaviour may achieve higher performance in beef cattle reproduction programs.

## 2 Material and Methods

### 2.1 Location and Animals

All procedures were approved by the ethics committee for animal use at Santo Amaro University, UNISA, São Paulo, Brazil (protocol number 34/2020), according to the Brazilian Council for the Control of Animal Experimentation (CONCEA).

The study was conducted at the Institute of Animal Science located in Sertãozinho, São Paulo, Brazil (21°07′19″ S; 48°00′08″ W). For the study, 16 healthy Nelore bulls were selected, with an average age of 24 months and an initial body weight of 654 kg, with no history of previous diseases, raised on pasture ( *Urochloa brizantha* ), with free access to water and mineral supplementation.

### 2.2 Experimental Design

All bulls (n=16) underwent two monthly evaluations (2 routines), which included temperament testing and a complete breeding soundness evaluation, including physical and reproductive tract examination as well as sperm evaluation.

Temperament was evaluated using Chute Score (CS) and Flight Speed (FS). Based on these results, bulls were classified into two groups: reactive (RE; *n* = 8) and calm (CA; *n* = 8). During both routines, semen samples were collected for laboratory evaluations, and blood samples were obtained for cortisol and testosterone analyses (Figure 1).

**FIGURE 1:**
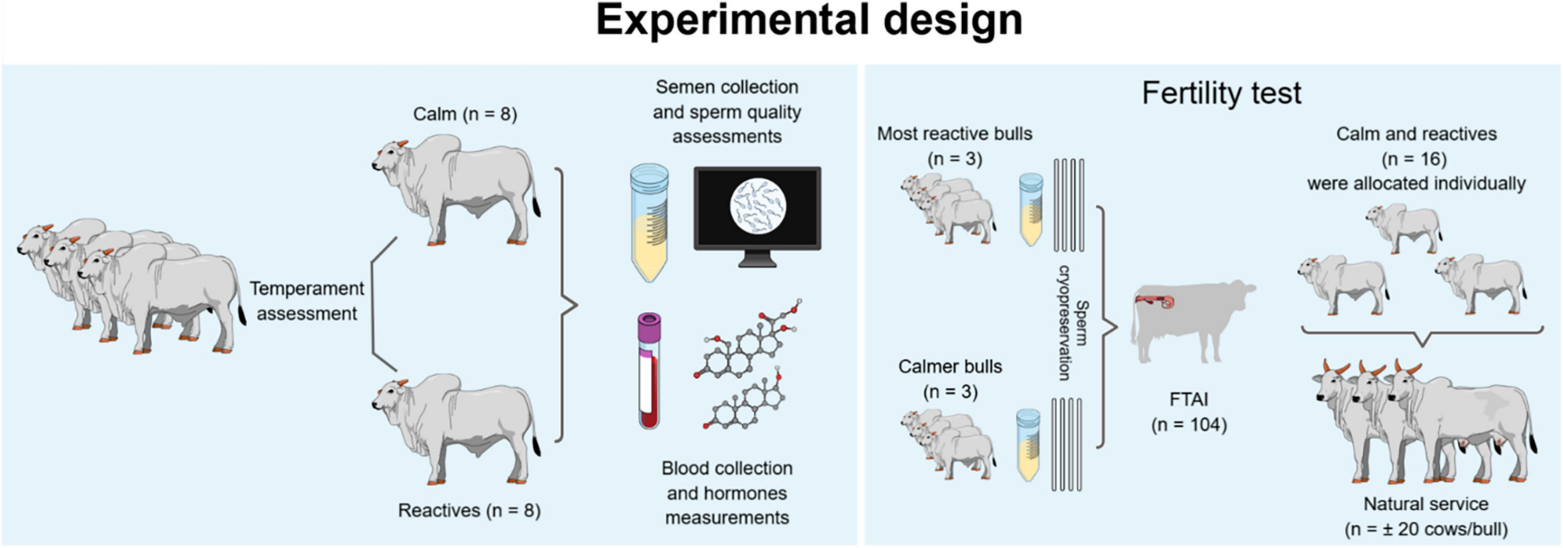
Schematic representation of the experimental design.

To establish maximum behavioural contrast and eliminate intermediate reactivity profiles, ejaculates from the three calmest and three most reactive bulls were selected to form two contrasting heterospermic pools for cryopreservation and subsequent use in fixed‐time artificial insemination (FTAI). Thirty days after FTAI and at the end of the natural service period, all cows enrolled in the experiment were examined by reproductive ultrasonography for pregnancy diagnosis.

Additionally, at the end of this routine, all 16 bulls underwent a natural service capacity test, being individually placed in paddocks with an average of 20 Nelore cows each for 90 days.

### 2.3 Temperament Assessment and Experimental Groups

To assess reactivity, the CS test was applied. The degree of disturbance in the animals was evaluated based on the frequency and vigour with which they moved within the containment chute for a period of 2 min. Each parameter assessed was related to a score ranging from 1 to 4, and in the end a sum was calculated, resulting in an agitation score ranging from 4 to 16 according to Braz et al. (2020). Based on the ethological assessment, individuals were classified as either calm (CA), receiving scores from 4 to 7 or reactive (RE), receiving scores from 8 to 16.

Additionally, FS from the containment chute was measured (Silva et al. 2025). For this test, the time spent by the animal to cover a straight‐line distance of 3 m from the opening of the containment chute door was recorded using a stopwatch. The average speed was calculated by dividing the distance covered by the time taken for each animal to complete the course. The principle behind this test is that naturally calmer and more docile animals take a longer time to leave the containment chute after being released (Silveira et al. 2006). To eliminate inter‐observer variability and ensure consistency, all temperament evaluations across both handling routines were performed by the same trained observer, who was blinded to individual animal scores during the second routine.

### 2.4 Blood Sample Collections and Hormonal Analysis

Blood samples for cortisol and testosterone determination were collected monthly via coccygeal vein puncture using a Vacutainer system and 10 mL vacuum tubes with clot activator. All blood collections were conducted in the morning, with a maximum interval of up to 3 h between the first and last animals to be collected. All samples were centrifuged for 15 min at 5000 rpm. After centrifugation, the serum was divided into aliquots and stored in mL disposable Eppendorf tubes and stored at −18°C until analysis.

Sorological assays for cortisol and testosterone in both groups were performed using the Milliplex Multi‐Species Hormone Magnetic Bead Panel kit (Merck KGaA, Darmstadt, Germany). Hormone quantifications were conducted according to Silva et al. (2025), and data were analyzed using Milliplex Analyst 5.0 software (Merck Millipore, Darmstadt, Germany). Intra and inter‐assay coefficients of variation for cortisol and testosterone were < 10%.

### 2.5 Semen Collection and Sperm Analysis

Semen collections were conducted monthly using electroejaculation (Neovet Autojac V3, Uberaba, Brazil) in automatic mode according to Silva et al. (2023). For each collection, a sterile 15 mL graduated tube attached to a disposable plastic funnel was used. Sperm kinetics were assessed using a computer‐assisted semen analysis system (CASA, Ivos Ultimate, version 14.0, Hamilton Thorne Inc., Beverly, USA). The following parameters were evaluated: total sperm motility (TM); progressive sperm motility (PM); straight‐line velocity (VSL); average path velocity (VAP); curvilinear velocity (VCL); amplitude of lateral head displacement (ALH); beat‐cross frequency (BCF); straightness (STR); linearity (LIN) and the proportion of rapid spermatozoa (RAP) according to the setup described by Dias et al. (2018).

Additionally, semen aliquots were fixed in 2% buffered saline formalin pre‐warmed to 36°C for subsequent evaluation of sperm morphology, using phase‐contrast microscopy at 1000× magnification. For this analysis, 100 sperm cells were counted, and defects were classified as major, minor and total (Blom 1973).

### 2.6 Sperm Cryopreservation

Semen samples collected during the second routine from the three most reactive bulls and the three calmest bulls were used to produce two heterospermic pools, according to each temperament group. Prior to pool formulation, each individual ejaculate from the six selected bulls was verified to meet standard andrological thresholds established by the Brazilian College of Animal Reproduction (CBRA 2013), displaying equivalent baseline laboratory quality (minimum of 50% total motility and total morphological defects <30%) across groups before pre‐dilution and combination.

To produce the pools, each ejaculate was individually evaluated for sperm concentration (Photometer SDM 1, Minitube, Tiefenbach, Germany) and pre‐diluted in egg yolk‐based extender (BotuBov, Botupharma, Botucatu, Brazil) to achieve a standardised sperm concentration of 60 × 10^6^ spermatozoa/mL. The pre‐diluted ejaculates were then combined in equal sperm proportions in their entirety and gently homogenised to form the final heterospermic pool. After pool formation, the samples were packaged in 0.5 mL straws (IMV Technologies, L'Aigle Cedex, France) and transferred to a programmable digital refrigerator (Minitube, Tiefenbach, Germany) for stabilisation at 5°C for 4 h. Freezing was carried out in an automated programmable freezing machine TK 3000 (Celeghini et al. 2008; TK Freezing Technology, Uberaba, Brazil). After this period, the samples were directly immersed in liquid nitrogen and stored in a cryobiological tank for subsequent laboratory quality analysis.

To assess the semen quality the straws were thawed in a water bath at 36°C for 30 s, homogenised in 1.5 mL Eppendorf tubes, which were kept in a dry water bath at a constant temperature of 35°C previously to sperm analysis. Post‐thaw semen samples were subjected to kinetic evaluation using a CASA system, according to the same parameters assessed for fresh semen. Subsequently, the samples were evaluated for plasma membrane integrity using fluorescence microscopy according to Silva et al. (2025). For this analysis, a 20 μL aliquot of semen was diluted in a microtube containing 20 μL of phosphate buffered saline (PBS) solution and 45 μL of fluorescent probe (1 mL of PBS solution, 35 μL of carboxyfluorescein diacetate and 120 μL of propidium iodide). The samples were evaluated after 10 min of incubation at 36°C in a dry bath, with 200 cells counted per sample.

### 2.7 Fertility Test

#### 2.7.1 Fixed‐Time Artificial Insemination

Semen pool straws from each experimental group were stored for artificial insemination. For the fixed‐time artificial insemination (FTAI), 104 multiparous, non‐lactating Nelore cows were selected and divided into two equal groups. They were randomly inseminated with semen pools from each group during the breeding season in south hemisphere. On the first day of the FTAI protocol, all cows were assessed for body condition using a scale from 1 to 5 (1 = emaciated, 5 = obese, according to Ayres et al. 2009). All FTAI protocols were conducted on the same farm during the regular breeding season (December to February) for beef cattle in Brazil.

Estrus synchronisation protocol followed the recommendations of Bo et al. (2019) for beef cattle, using hormones from OuroFino Saúde Animal (Cravinhos, São Paulo, Brazil). All cows received a 1 g progesterone intravaginal implant (Sincrogest) on the first day of the protocol (D0) along with the application of 2 mg of estradiol benzoate (Sincrodiol). After 8 days (D8), all animals received an injection of 0.52 mg of sodium cloprostenol (Sincrocio), 1 mg of estradiol cypionate (SincroCP), 400 IU of equine chorionic gonadotropin (Sincro eCG) and the progesterone implant was removed. The FTAI was performed in all females 48–54 h after the progesterone implant withdrawal.

#### 2.7.2 Natural Service Test

All 16 bulls initially selected were eligible for sexual behaviour and performance assessment. For this phase, each bull was individually allocated to a paddock of approximately 60,000 m^2^ composed of *Brachiaria decumbens* , with 20 multiparous Nellore cows (mean age: 64.79 months; mean body weight: 427.87 kg) assigned to each bull (1:20 bull‐to‐cow ratio) for a 90‐day breeding period.

At the end of the breeding season, the bulls were removed from the female groups and pregnancy diagnosis was performed by reproductive ultrasonography.

### 2.8 Statistical Analysis

The effect of stress on the productive and reproductive performance of bulls was evaluated using the linear model of analysis of variance with repeated measures over time (PROC MIXED) using the SAS software (SAS Institute Inc., Cary, USA). The statistical model included the fixed effect of reactivity category (Calm and Reactive), the assessment routine and their interaction. The residual variance was modelled considering the best (co)variance structure according to the Akaike information criterion. Each variable was tested for normality and homogeneity, and variables that did not meet these criteria were log‐transformed before statistical analysis.

Additionally, the results of the behavioural assessments (ethogram and escape velocity from the containment chute) were considered as independent variables in a regression model, testing the degree of dependence of each reproductive variable studied on the different behavioural patterns exhibited by the bulls.

The conception rate in FTAI and after the natural mating test was evaluated using a logistic regression model (PROC GLIMMIX, SAS) and binomial response involved in the ratio of the number of successes to the number of events. The conception rate was considered the dependent variable, while the cows' body condition score, escape velocity from the containment chute, animal reactivity level and their potential interactions were considered independent variables. For the pregnancy rate after natural mating, the individual bull effect was also included as a random effect in the model.

## 3 Results

### 3.1 Behavioural Assessment

Significant differences in CS were observed between groups when analysed independently of the experimental routine, with reactive bulls showing higher agitation scores compared with calm bulls (8.60 ± 0.34 vs. 4.50 ± 0.38 for RE and CA, respectively; *p* < 0.0001). Similar results were obtained when the comparison was performed within each handling routine, where reactive bulls consistently displayed greater agitation (Table 1).

**TABLE 1:** Mean (± SD) chute score (CS) and flight speed (FS) evaluated for bulls considered as calm (CA) or reactive (RE), according to the different experimental routines.

Routine	Temperament evaluation	CA	RE	*p*
R1	CS	4.75 ± 0.64^a^	10.40^b^ ± 0.57^b^	0.0003
	FS	2.34 ± 0.84	1.26 ± 0.68	0.34
R2	CS	4.25 ± 0.64^a^	6.80 ± 0.57^b^	0.02
	FS	3.71 ± 1.03	2.84 ± 0.68	0.49

*Note:* ^a,b^Different letters in the same line indicate significant differences.

No differences were detected in FS when groups were compared independently of the routine (3.02 ± 0.64 vs. 2.05 ± 0.46 for RE and CA bulls, respectively; *p* = 0.24). Likewise, no differences were observed when FS was analysed within each routine (Table 1).

### 3.2 Fresh Semen Analysis

All semen samples collected met the minimum quality standards established for bovine semen, according to the reference parameters proposed by the Brazilian College of Animal Reproduction (CBRA 2013). No effects of routines or animal temperament were observed on the sperm kinetic parameters evaluated in fresh semen within each experimental group (Table 2; *p* > 0,05). Similarly, no differences were observed in the quantification of morphological defects when comparing bulls with contrasting temperaments.

**TABLE 2:** Mean (±) sperm quality evaluated for fresh semen of bulls considered as calm (CA) or reactive (RE).

Variables	Groups			
	CA	RE	SD	*p*
TM (%)	90.62	75.70	12.55	0.27
PM (%)	68.25	55.40	10.14	0.24
VAP (μm/S)	100.91	106.53	13.97	0.69
VSL (μm/S)	80.93	84.16	8.63	0.72
VCL (μm/S)	168.65	184.47	28.68	0.59
ALH (μm)	6.27	7.36	1.02	0.32
BCF (μm)	31.70	28.60	2.48	0.25
STR (%)	81.65	80.80	3.47	0.81
LIN (%)	51.62	49.60	4.01	0.62
FAST (%)	87.75	72.50	12.98	0.27
Major defects (%)	10.79	13.4	3.41	0.45
Minor defects (%)	10.32	9.9	3.29	0.91
Total defects (%)	21.42	23.3	4.97	0.79

*Note:* Values represent data from a total of 32 ejaculates (16 ejaculates per group: *n* = 8 bulls per group across two handling routines).

Abbreviations: ALH, amplitude of lateral head displacement, μm; BCF, beat cross frequency, Hz; FAST, fast‐moving sperm, %; LIN, linearity, %; PM, progressive motility, %; SD, standard deviation; TM, total motility, %; VAP, average path velocity, μm/s; VCL, curvilinear velocity, μm/s; VSL, straight linear velocity, μm/s; STR, straightness, %.

### 3.3 Post‐Thaw Semen Analysis

No effect of temperament was observed on any sperm kinetic parameters in post‐thaw semen assessment (Table 3). Likewise, no interactions were detected between the effect of the routines and the evaluated kinetic variables.

**TABLE 3:** Mean (±) sperm quality evaluated for post‐thaw semen of bulls considered as calm (CA) or reactive (RE).

Variables	Groups			
	CA	RE	SD	*p*
TM (%)	37.81	28.16	6.86	0.20
PM (%)	20.25	15.04	3.29	0.15
VAP (μm/S)	52.46	48.82	2.56	0.19
VSL (μm/S)	38.18	36.21	2.15	0.38
VCL (μm/S)	84.17	81.53	5.20	0.62
ALH (μm)	3.45	3.38	0.25	0.78
BCF (μm)	9.07	9.88	0.47	0.13
STR (%)	72.26	74.26	2.72	0.48
LIN (%)	44.88	44.40	2.79	0.86
FAST (%)	22.95	15.31	3.97	0.09
PMI (%)	23.62	18.5	3.94	0.28

*Note:* Evaluations were performed on heterospermic pools produced by combining ejaculates from three selected bulls per group (*n* = 3 ejaculates per pool/group).

Abbreviations: ALH, amplitude of lateral head displacement, μm; BCF, beat cross frequency, Hz; FAST, fast‐moving sperm, %; LIN, linearity, %; PM, progressive motility, %; PMI, plasma membrane integrity, %; SD, standard deviation; STR, straightness, %; TM, total motility, %; VAP, average path velocity, μm/s; VCL, curvilinear velocity, μm/s; VSL, straight linear velocity, μm/s.

Similarly, no differences in plasma membrane integrity were observed when groups were compared independently of routine (18.50 ± 2.92 and 23.62 ± 3.26 for RE and CA bulls, respectively; *p* = 0.28). No effects were detected within each experimental routine.

### 3.4 Hormonal Analysis

No differences in cortisol concentrations were observed when compared to the groups with contrasting temperament independently of the routines (10.90 and 10.57 ng/mL for RE and CA, respectively; *p* = 0.82). However, RE bulls exhibited higher mean cortisol concentrations during the second routine (Table 4).

**TABLE 4:** Mean (± SD) cortisol (ng/mL) and testosterone (ng/mL) concentrations quantified for bulls considered as calm (CA) or reactive (RE), according to the different experimental routines.

Routine	Cortisol (ng/mL)			*p*	Testosterone (ng/mL)			*p*
	CA	RE	SD		CA	RE	SD	
1	6.08	14.40	4.50	0.14	7.28	9.66	5.81	0.31
2	3.46^a^	20.17^b^	5.81	0.01	11.25	4.91	6.31	0.32

*Note:* Different letters in the same line indicate significant differences.

No effect of temperament was detected on mean testosterone concentrations, whether analyzed independently of routine (8.16 and 21.86 ng/mL for RE and CA, respectively; SD = 5.37; *p* = 0.14) or within each experimental routine.

### 3.5 Pregnancy Rates

No effect of temperament was observed on pregnancy rates in cows subjected to FTAI using pooled semen from the calm or reactive bulls. Pregnancy rates were 46.15% and 42.31% for cows inseminated with semen from CA and RE bulls, respectively (*p* = 0.42; Figure 2).

**FIGURE 2:**
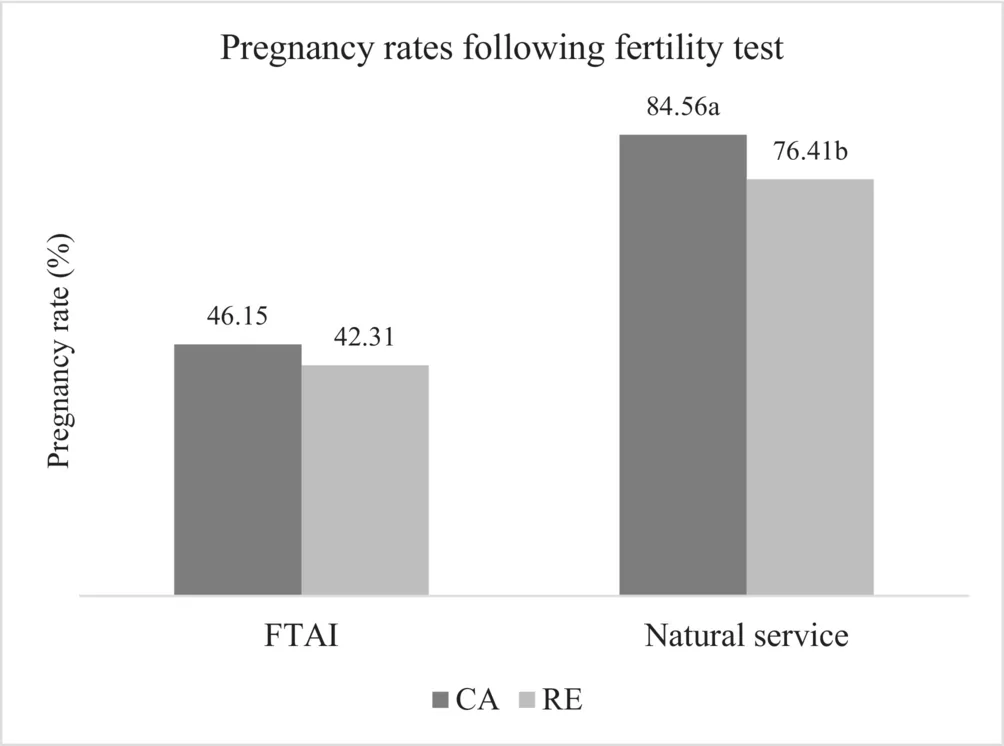
Mean (±) pregnancy rates following fertility tests including calm (CA) and reactive (RE) bulls in fixed‐time artificial insemination (FTAI; *p* = 0.42) and natural service (*p* = 0.0001).

However, cows allocated to natural service with CA bulls exhibited higher pregnancy rates compared with those allocated to RE bulls (*p* = 0.0001; Figure 2), indicating reduced reproductive efficiency in reactive bulls.

## 4 Discussion

Numerous studies have investigated the impact of temperament on livestock production, consistently associating reactive temperaments with adverse outcomes such as reduced weight gain (Voisinet et al. 1997), lower meat quality (Sant'anna et al. 2019) and decreased milk yield (Orbán et al. 2011). Within this context, reproductive efficiency is regarded as one of the most critical traits in livestock production systems, and the detrimental effects of reactivity have been well documented in female cattle (Cooke et al. 2017; Guerson et al. 2021).

Temperament is defined as the individual pattern of behaviour that remains consistent over time and across different situations (McMahon et al. 2023). In cattle, temperament is often classified based on the degree of docility or aggressiveness (i.e., calm vs. reactive; Mello et al. 2020; Silva et al. 2025). Reactive animals are commonly associated with elevated serum cortisol concentrations (Cooke 2014; Braz et al. 2020), likely due to a heightened and more frequent activation of the hypothalamic–pituitary–adrenal (HPA) axis in response to environmental or handling stressors (Curley et al. 2008). Consistent with this, in the present study, bulls classified as reactive (RE) exhibited higher serum cortisol concentrations compared to calm (CA) group during the second routine.

Despite the clear association between reactivity and cortisol secretion, this behavioural profile does not appear to affect the androgenic endocrine axis. In the present study, testosterone concentrations did not differ between temperament groups, either independently of handling routine or when evaluated within each routine. These results agree with previous findings showing that acute increases in glucocorticoids do not necessarily suppress testosterone production in bulls under normal physiological conditions (Lockwood et al. 2017; Braz et al. 2020; Silva et al. 2025). This suggests that, although reactive animals may experience greater HPA axis activation during stressful events, the extent or duration of this response might not be sufficient to impair Leydig cell function or steroidogenesis in mature bulls.

When evaluating the relationship between temperament, endocrinology and reproductive parameters in young bulls, Lockwood et al. (2017) reported that reactive animals exhibited a higher percentage of sperm defects compared to less reactive individuals. Similarly, Silva et al. (2025) observed a greater proportion of minor defects in reactive young bulls compared to calm ones. In the present study, however, no effect of reactivity was found on any semen quality parameter for both raw and post‐thaw semen, suggesting that young bulls may be more susceptible to temperament‐related impacts on semen quality—an effect not observed in studies involving adult bulls (Braz et al. 2020). Furthermore, the preserved functionality of the hypothalamic–pituitary–gonadal axis, evidenced by normal testosterone production across both groups, may be linked to the maintenance of normal spermatogenesis, thereby preventing reactive bulls at sexual maturity from experiencing impairments in semen quality.

As demonstrated by previous studies (Cooke et al. 2011, 2017; Rueda et al. 2015), reactivity observed for female cattle exerts a negative influence on pregnancy rates after artificial insemination (AI). According to Vedovatto et al. (2021), reactive cows tend to develop smaller dominant follicles, and consequently, smaller corpus luteum diameters and lower progesterone production, which may justify the worse performance in AI. In bulls, AI is considered one of the most sensitive methods for assessing semen fertility potential. In this context, although a low number of females was used in FTAI, few available studies have evaluated the effect of temperament on bovine semen fertility in artificial insemination programs. In the present study, no differences were observed in conception rates following FTAI using semen pools from bulls with contrasting temperament. These findings are consistent with the equivalence observed between the groups regarding post‐thaw kinetic and functional sperm parameters, which was also reflected in similar pregnancy rates following FTAI.

Although artificial insemination is a key reproductive tool worldwide, its adoption in Brazil remains limited (about 25% of cows in reproductive age in the country are inseminated annually), with most cows bred by natural service (ASBIA 2025). Thus, natural service still plays a crucial role in a large number of cattle production countries, highlighting the importance of evaluating bull fertility and mating efficiency under field conditions. Male sexual behaviour is a major determinant of reproductive success, as fertility relies not only on semen quality but also on libido, mating ability and the capacity to detect and mount estrous females (Le Danvic et al. 2015).

Male sexual behaviour, encompassing libido (sexual drive) and serving capacity (the ability to seek, mount and service estrous females), is a primary determinant of bull fertility under natural mating conditions (Pearson 2025). Classical studies have demonstrated that bulls with high serving capacity achieve higher pregnancy rates and tighter calving windows compared to low‐capacity bulls, independent of seminal quality (Blockey 1978; Chenoweth 1997). However, bull sexual behaviour can vary substantially depending on age and sexual experience (Chenoweth 1983), social dominance hierarchy (López et al. 1999) and environmental conditions.

To our knowledge, this is the first study to evaluate the breeding capacity and fertility of reactive bulls in natural service. Lower conception rates in natural breeding were observed for the RE group (76%) compared to CA (84%). Although no differences were observed in fresh semen quality between the groups, behavioural characteristics may justify the lower rates observed for reactive animals. In this context, reactive cattle often remain in a state of alert (Estévez‐Moreno et al. 2022) due to their inability to deal with the environment and the constant feeling of fear and insecurity (Boissy et al. 2005).

Although sexual behaviour parameters, such as mounting frequency and libido scores, were not directly monitored during the 90‐day pasture breeding period due to logistical constraints, according to Fraser (1959), bulls classified as extremely apprehensive, a trait associated with a reactive temperament, exhibited significantly longer latencies to copulate with cows in estrus than bulls from other temperament categories. Thus, reactive bulls may allocate more energy towards vigilance and avoidance behaviours rather than courtship and mating, potentially reducing the frequency and efficiency of intromissions and successful copulations. This behavioural shift may explain the lower pregnancy outcome observed in the RE group, despite similar semen quality.

## 5 Conclusion

Reactive bulls exhibit higher cortisol concentrations during routine handling, but no significant effect on testosterone secretion or on the evaluated semen quality parameters was detected in either fresh or post‐thaw samples. Nevertheless, under natural service in field conditions, reactive bulls achieved lower pregnancy rates compared with calm bulls, suggesting that behavioural factors associated with reactivity may compromise mating efficiency despite equivalent seminal quality and endocrine status.

## Author Contributions

**Larissa de Almeida Martin:** data curation, investigation, visualisation, writing – original draft. **Mariana Karla Francolino da Silva:** data curation, visualisation, writing – original draft, writing – review and editing. **Marcelo Sant’Ana Borges:** data curation, formal analysis, validation. **Luana Gomes Fernandes:** investigation. **Richardt Gama Landgraf:** investigation. **Fábio Morato Monteiro:** resources, investigation. **André Maciel Crespilho:** conceptualization, investigation, funding acquisition, resources, methodology, project administration, supervision, writing – review and editing.

## Funding

This work was supported by the Fundação de Amparo à Pesquisa do Estado de São Paulo (2019/06456‐2).

## Conflicts of Interest

The authors declare no conflicts of interest.

## Data Availability

The data that support the findings of this study are available from the corresponding author upon reasonable request.
